# *Cyanidioschyzon merolae* aurora kinase phosphorylates evolutionarily conserved sites on its target to regulate mitochondrial division

**DOI:** 10.1038/s42003-019-0714-x

**Published:** 2019-12-20

**Authors:** Shoichi Kato, Erika Okamura, Tomoko M. Matsunaga, Minami Nakayama, Yuki Kawanishi, Takako Ichinose, Atsuko H. Iwane, Takuya Sakamoto, Yuuta Imoto, Mio Ohnuma, Yuko Nomura, Hirofumi Nakagami, Haruko Kuroiwa, Tsuneyoshi Kuroiwa, Sachihiro Matsunaga

**Affiliations:** 1 Department of Applied Biological Science, Faculty of Science and Technology, Tokyo University of Science, Noda, Chiba 278-8510 Japan; 2 Research Institute for Science and Technology, Tokyo University of Science, Noda, Chiba 278-8510 Japan; 3 RIKEN Center for Biosystems Dynamics Research, 3-10-23 Kagamiyama, Higashi-Hiroshima, Hiroshima 739-0046 Japan; 4 Department of Cell Biology, Johns Hopkins University School of Medicine, 725N. Wolfe Street, 100 Biophysics, Baltimore, MD 21205 USA; 5 National Institute of Technology, Hiroshima College, Hiroshima, 725-0231 Japan; 6 RIKEN CSRS, 1-7-22 Suehiro-cho, Tsurumi-ku, Yokohama, Kanagawa 230-0045 Japan; 7 Protein Mass Spectrometry Group, Max Planck Institute for Plant Breeding Research, Carl-von-Linne-Weg 10, 50829 Cologne, Germany; 8 Department of Chemical and Biological Science, Japan Women’s University, Tokyo, 112-8681 Japan

**Keywords:** Cell biology, Plant sciences

## Abstract

The mitochondrion is an organelle that was derived from an endosymbiosis. Although regulation of mitochondrial growth by the host cell is necessary for the maintenance of mitochondria, it is unclear how this regulatory mechanism was acquired. To address this, we studied the primitive unicellular red alga *Cyanidioschyzon merolae*, which has the simplest eukaryotic genome and a single mitochondrion. Here we show that the *C. merolae* Aurora kinase ortholog CmAUR regulates mitochondrial division through phosphorylation of mitochondrial division ring components. One of the components, the Drp1 ortholog CmDnm1, has at least four sites phosphorylated by CmAUR. Depletion of the phosphorylation site conserved among eukaryotes induced defects such as mitochondrial distribution on one side of the cell. Taken together with the observation that human Aurora kinase phosphorylates Drp1 in vitro, we suggest that the phosphoregulation is conserved from the simplest eukaryotes to mammals, and was acquired at the primitive stage of endosymbiosis.

## Abstract (web summary)

Kato et al. show that red alga *Cyanidioschyzon merolae* aurora kinase CmAUR regulates mitochondrial division by phosphorylating mitochondrial division ring components. Depletion of the evolutionarily conserved phosphorylation site on CmAUR targets causes defective mitochondrial divisions, suggesting an ancient origin of this regulation during endosymbiosis.

## Introduction

Mitochondria are organelles that play important roles in energy production and several metabolic pathways in eukaryotic cells, and are thought to be derived from a sole alphaproteobacterium that invaded the protoeukaryote^1,2^. The endosymbiosis of mitochondria is thought to be crucial for the evolution of eukaryotic cells^3^. Because the machinery regulating mitochondrial division, such as a mitochondrial division ring, is well conserved among eukaryotes, it can be predicted that the mechanism, which regulates mitochondrial division was acquired in the process of establishing this endosymbiosis^3–5^.

Some nuclear-encoded genes derived from the ancestor host cell, such as *Drp1*, *Opa1*, and *Fis1*, control mitochondrial morphology in animal cells^6^. Drp1 forms a mitochondrial division ring at the mitochondrial division site. Mitochondrial division is induced by contraction, which is caused by a structural change in Drp1. Drp1 recruitment to mitochondria depends on an integral membrane protein, Fis1, which is located in the mitochondrial outer membrane^6^. In addition, the mitochondrial outer membrane protein Mff recruits Drp1 to mitochondria independently of Fis1^6^. The dynamin-related protein Opa1, which is localized to the mitochondrial inner membrane, induces mitochondrial fusion^6^. In animal, yeast and plant cells, regulation of mitochondrial fragmentation in the G2/M phase by mitotic kinases is required for the proper distribution of mitochondria to daughter cells^6^. Previous studies using human cultured cells have revealed that mitochondrial proliferation occurs before mitosis, and that this phenomenon is controlled by phosphoregulation of Drp1 by cyclin-dependent kinase1^7^. The mitochondrial localization of Drp1 is regulated by Aurora A via phosphorylation of RalA, which belongs to the Ras family, part of the small GTPase superfamily^8^. Phosphorylated RalA forms a protein complex with Drp1, RalBP1, Cyclin B, and CDK1, which is recruited to the mitochondrial outer membrane in a Mff-dependent manner^8^.

*Cyanidioschyzon merolae* is an ultra-small (> 2 µm in diameter) unicellular red alga that inhabits hot and sulfate-rich springs. *C. merolae* has a simple cell structure, with a single mitochondrion and a single plastid^9,10^. The mitochondrion divides only once per cell cycle, during M phase^9–11^. These features would enable us to investigate the mitochondrial division precisely. In addition, sequencing of the *C. merolae* genome and the development of molecular genetic analysis methods have made it possible to investigate detailed molecular mechanisms in this organism^12–16^. Therefore, we assumed that *C. merolae* is the best model for investigating the molecular mechanisms of coordination between mitochondria and host cells.

Aurora kinases are members of a highly conserved mitotic kinase family, which controls mitotic spindle formation and chromosome segregation^17–22^. Although many other eukaryotes have two or three Aurora kinases, *C. merolae* has only one Aurora kinase gene (*CmAUR*) in its genome^23^. Previously, we revealed that a green fluorescent protein (GFP) fusion protein of CmAUR in *C. merolae* was localized to the spindle and the mitochondrion^23^. Localization to the mitochondrion was observed throughout the cycle, and CmAUR-GFP was especially accumulated at the mitochondrial division site in M phase, which is the only phase when mitochondrial division occurs in *C. merolae*^23^. Its localization to the mitochondrion was shown to be regulated by a kinesin-like, division-inducing protein (TOP)^24^. Given these findings, we presumed that CmAUR regulates mitochondrial division during M phase.

To test this hypothesis, here, we investigated whether regulation of mitochondrial division by a mitotic kinase occurs in primitive eukaryotic cells using *C. merolae*. Our results indicate that Aurora kinase regulates mitochondrial division in G2/M phase through phosphorylation of the Drp1 ortholog CmDnm1. Our results also show that human Aurora kinases directly phosphorylate Drp1 in vitro. These results suggest the conservation of this mechanism among eukaryotes. The mechanism may have been acquired in common ancestral cells and may have contributed to the establishment of endosymbiosis.

## Results

### Evaluation of the Aurora kinase ortholog CmAUR

**Fig. 1:**
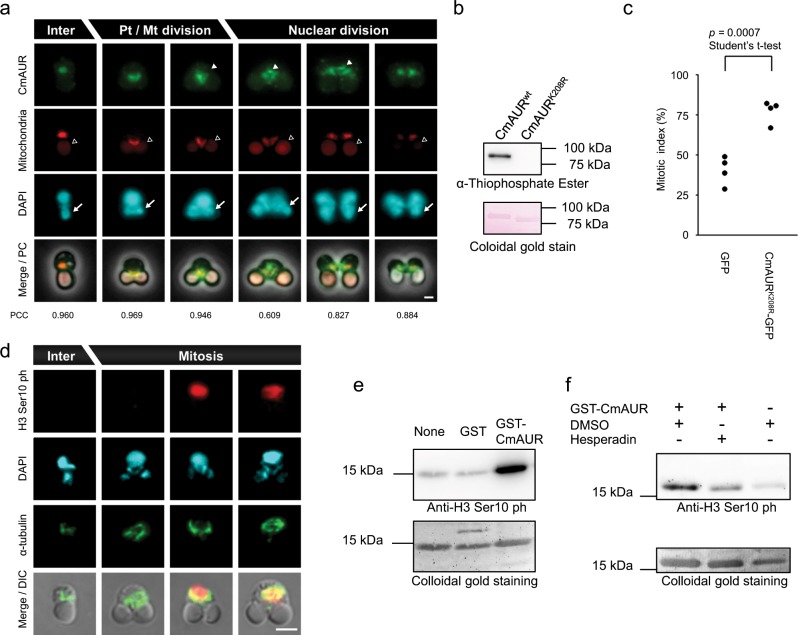
The *Cyanidioschyzon merolae* Aurora kinase CmAUR has conserved properties of Aurora kinase orthologs. **a** Localization of CmAUR by immunofluorescence analysis. CmAUR was stained with CmAUR antibody. Mitochondria were stained by Ef-Tu antiserum. DNA was stained with DAPI. White arrowheads indicate signals localized to the spindle or spindle pole. Black arrowheads indicate plastid autofluorescence. Arrows indicate plastid DNA. The Pearson correlation coefficient (PCC) in each cell was calculated using areas without plastids. We observed 24 cells and present representative images in this figure. **b** Autophosphorylation assay of wild-type and mutant CmAUR. ATP-γS was used as a substrate for autophosphorylation, and the phosphorylation was detected by western blotting using thiophosphate ester antibody. **c** Mitotic inhibition in a dominant-negative mutant of CmAUR. CmAUR^K208R^ is a kinase-dead mutant; GFP was used as a negative control. Relevant genes were transiently overexpressed in *C. merolae* and mitotic cells among transfectants were counted. The error bars indicate standard error of the mean. **d** Immunofluorescence image of histone H3 Ser10 phosphorylation in mitosis. More than 18 cells were observed. **e** In vitro phosphorylation assay of histone H3 Ser10 with recombinant CmAUR. Glutathione-*S-*transferase (GST) was used as a negative control. Distilled water was used as a blank sample. **f** In vitro phosphorylation assay of histone H3 Ser10 treated with hesperadin. The concentration of hesperadin was 10 µm. The concentration of dimethyl sulfoxide in the final solution was 0.1% (v/v). Bars: 1 µm **a**, 2  μm **c**.

Although some previous studies have focused on CmAUR localization in *C. merolae*^23,24^, little is known about its role as an Aurora kinase, such as in mitotic progression through histone H3 Ser10 phosphorylation in mitosis^25^. Thus, we considered that it was important to test the Aurora kinase function of CmAUR. First, we conducted immunostaining to determine the cellular localization of CmAUR. Previously, we reported a CmAUR antibody and confirmed its specificity^24^. The immunostaining showed that CmAUR localized to the mitotic spindle and mitochondrion (Fig. 1a, Supplementary Fig. 1). To investigate its function in cell cycle progression, we transiently expressed a kinase-dead version of CmAUR (CmAUR^K208R^) in *C. merolae*. This mutant of CmAUR was designed by reference to previous reports of kinase-dead human Aurora A^26^ and B^27^, and we confirmed that recombinant CmAUR^K208R^ protein had no autophosphorylation activity (Fig. 1b). Expression of CmAUR^K208R^ in *C. merolae* delayed M phase, and this was most likely caused by a dominant-negative effect, analogous to a previous study in human^27^ (Fig. 1c). These results suggested that CmAUR has an important role in M phase in *C. merolae*, like its orthologs in other eukaryotes. We also tested whether CmAUR has capability as a kinase of histone H3 Ser10. Similar to other species, *C. merolae* showed phosphorylation of histone H3 Ser10 during M phase, as reported previously^28^ (Fig. 1d, Supplementary Fig. 2). Recombinant glutathione-*S-*transferase (GST)-CmAUR strongly phosphorylated H3 Ser10 in vitro (Fig. 1e). Although a weak histone H3 phospho Ser10 band was detected in the GST only-treated sample, the intensity was not different from the band in the non-GST-treated sample (Fig. 1e). Therefore, we consider that H3 Ser10 phosphorylation by GST-CmAUR depends on CmAUR. For further confirmation of whether CmAUR works as an Aurora kinase, we treated GST-CmAUR in vitro with hesperadin, an Aurora kinase inhibitor. Phosphorylation of H3 Ser10 by GST-CmAUR was inhibited by hesperadin (Fig. 1f). In summary, these results suggest that CmAUR functions as an Aurora kinase.

### Relationship between mitochondrial division and CmAUR

**Fig. 2:**
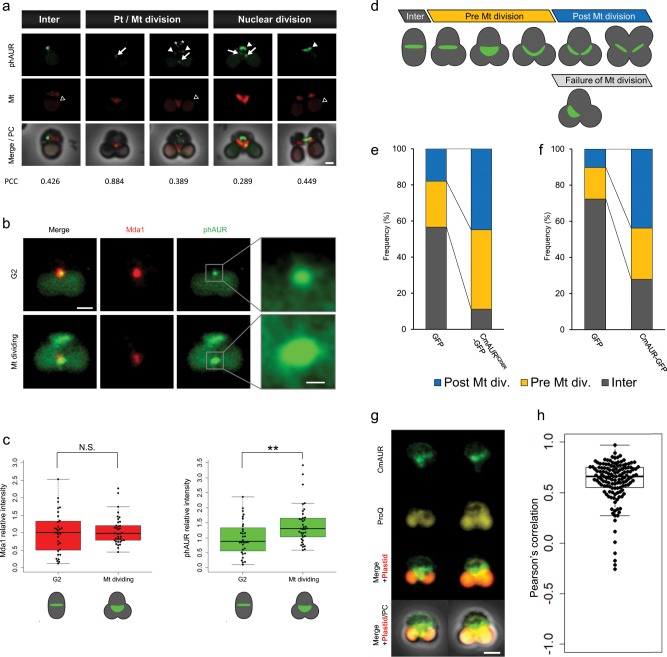
CmAUR involvement in mitochondrial division in mitosis. **a** Localization of activated CmAUR. Activated CmAUR was visualized using antibody to phosphorylated Aurora kinase. Mt indicates mitochondria, which were stained by Ef-Tu antiserum. Arrows indicate speckles localized to the mitochondrial division site. White arrowheads indicate signals that seemed to be localized to the spindle or spindle pole. Asterisks indicate speckles, which are not predicted to be localized to a specific organelle. Black arrowheads indicate plastid autofluorescence signals. Inter, interphase; Pt/Mt division, plastid/mitochondrial dividing phase; PC, phase contrast image. The PCC of each cell was calculated using areas without plastids. More than 30 cells were observed. **b** Colocalization of activated CmAUR and mitochondrial ring protein Mda1. More than 30 cells were observed. **c** Intensity of activated CmAUR speckles localized to the mitochondrial division ring. The relative intensity of human phospho-Aurora antibody (phAUR) and Mda1 in each cell is indicated on the right and left, respectively. **p *=* 0.0045 (two-sided Student’s *t* test). **d** Scheme of mitochondrial division in *C. merolae*. **e**, **f** Effect of kinase-dead mutant **e** and overexpression **f** of CmAUR on mitochondrial division in *C. merolae*. GFP was used as a negative control. GFP, CmAUR^K208R^-GFP, and CmAUR-GFP were transiently overexpressed in *C. merolae*. According to the classification in **d**, transfectants were counted. **g** Co-staining of CmAUR and with ProQ Diamond. CmAUR was stained with α-CmAUR antibody. The two images are of representative M-phase cells. The red autofluorescence signal indicates plastids. **h** Colocalization analysis of ProQ Diamond and CmAUR. Co-stained M-phase cells were analyzed. Each point indicates the PCC of individual *C. merolae* cells. The area in each cell without plastids was used for analysis. *n* = 135. Bars: 1 μm (**a**, **b**, left), 250 nm (**b**, right), 2 µm **g**.

To investigate the molecular processes regulated by CmAUR, we conducted immunostaining analyses of autophosphorylated CmAUR. Previous studies suggested that Aurora kinase activates itself through autophosphorylation^29,30^. Because the self-phosphorylation site of Aurora kinase is well conserved among Aurora kinase orthologs, a commercial human phospho-Aurora (phAUR) antibody was able to detect self-phosphorylated CmAUR (Supplementary Fig. 3a–d). By using the phAUR antibody, we demonstrated that activated CmAUR partially localized to the mitochondrion (Fig. 2a, Supplementary Fig. 4). Co-immunostaining using phAUR and an antibody against the mitochondrial division ring component Mda1 revealed that phAUR was localized to the mitochondrial division ring (Fig. 2b). To investigate whether the localization changed depending on cell cycle progression, we analyzed phAUR and Mda1 in both G2 and M phase. The intensity of the phAUR signals on the mitochondrial division ring increased as mitochondrial division progressed (Student’s *t* test, *p* < 0.01) but not Mda1 signals (Fig. 2b, c). Because Mda1 is a protein that accumulates on the mitochondrial division ring, this result suggests that the phAUR signals increased independently of the accumulation of other mitochondrial division-related proteins in M phase such as Mda1. After mitochondrial division, the phAUR signal was observed on structures other than the mitochondrion (Fig. 2a, white arrowheads). Considering the morphological features and a previous report on *C. merolae* mitosis^31^, we deduced that this structure was the mitotic spindle. However, localization on speckles, which are clearly different from the spindle, was also observed. This suggests that CmAUR does not co-localize exclusively with mitochondria, as already reported in human Aurora A^25^. We conclude that CmAUR activity is involved in both mitochondrial division and mitotic spindle formation.

To confirm the role of CmAUR in mitochondrial division during mitosis, we overexpressed both wild-type and kinase-dead type CmAUR. Then we measured the frequency of each phase of the transformants (interphase, pre-mitochondrial division phase, and post-mitochondrial division phase) in the mitochondrial replication process (Fig. 2d). The transformants overexpressing both CmAUR^K208R^ and wild-type CmAUR were arrested at the pre- and post-mitochondrial division phases (Fig. 2e, f; Supplementary Tables 1, 2). Because the proportion of wild-type and CmAUR^K208R^ overexpressing cells in each phase did not seem to be different, it is not clear whether the kinase activity of CmAUR had an impact on the mitochondrial division. However, these data indicate that CmAUR kinase activity and/or another CmAUR function can regulate both nuclear and mitochondrial division. Division of the mitochondrion occurs before nuclear division in *C. merolae*^31^. Thus, the inhibition of mitochondrial division was most likely not caused by nuclear division.

To predict the localization of substrates phosphorylated by CmAUR in vivo, we conducted co-staining for CmAUR and with ProQ diamond. ProQ diamond binds total phosphorylated proteins and we previously demonstrated that ProQ diamond staining is effective in *C. merolae*^24^. In co-stained M-phase *C. merolae* cells, CmAUR signals were partially co-localized with ProQ signals (Fig. 2g, h). Because CmAUR was localized to the mitochondrion (Fig. 1a, Supplementary Fig. 1), this result implies that CmAUR phosphorylates substrates in mitochondria.

### Identification of the substrates of CmAUR

**Fig. 3:**
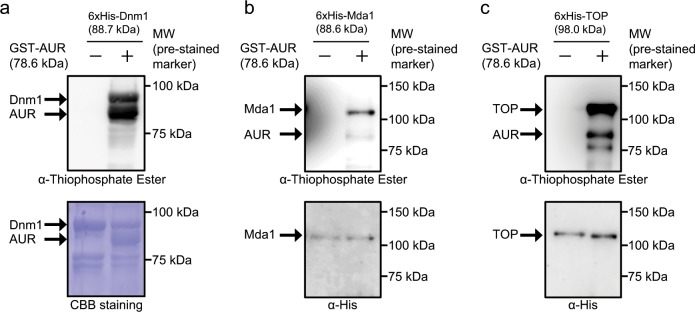
CmAUR phosphorylated mitochondrial division-related proteins in vitro. **a**–**c** In vitro kinase assays of recombinant CmAUR with recombinant mitochondrial division ring proteins. CBB staining indicates Coomassie Brilliant Blue staining.

Because localization analysis of phAUR suggested a relationship between mitochondrial division and CmAUR, we predicted that CmAUR is involved in mitochondrial division through phosphorylation of mitochondrial division ring components. To verify this presumption, we performed in vitro kinase assays using recombinant CmDnm1, Mda1, and TOP, which are known to be involved in mitochondrial division in *C. merolae*^11,24,32^. CmDnm1 is a highly conserved protein among eukaryotes and an orthologue of human Drp1 (DNM1L), which forms a ring around mitochondria and induces their division in eukaryotes such as mammalian, nematode, and yeast cells^33–35^. In these assays, CmAUR phosphorylated CmDnm1, Mda1, and TOP (Fig. 3a–c). These results suggested that CmAUR regulates mitochondrial division through phosphorylation of mitochondrial division ring components in *C. merolae*.

### Determination of phosphorylation sites of CmDnm1 by CmAUR

**Fig. 4:**
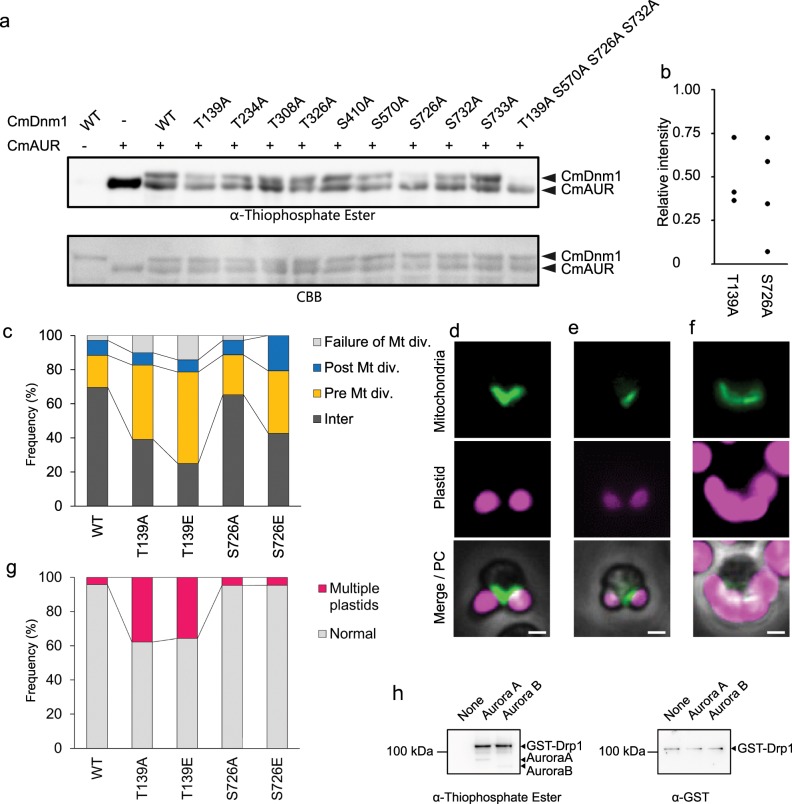
Effects of amino-acid substitutions of phosphorylation sites of Dnm1 by Aurora kinase in Dnm1 on mitochondrial division. **a** In vitro kinase assay of recombinant CmAUR and site-directed Dnm1 mutants and those with mutations of 4, 7, 8, and 9 residues (see Supplementary Table 3). **b** Quantification of in vitro kinase assays of CmDnm1 single amino-acid variants. Signals from different membranes were normalized against the luminosity of wild-type CmDnm1. The relative value of wild-type CmDnm1 was 1. *n* = 3 for T139A, *n* = 4 for S726A. The error bars indicate standard error of the mean. **c** Frequency of transformants expressing CmDnm1 variants. Alanine and phosphomimetic glutamic acid mutants of CmDnm1^T139^ and CmDnm1^S726^ were overexpressed in *C. merolae* cells. According to classification Fig. 2d, transfectants were counted. Transfectants with an aberrant number of plastids (three or more) were not counted. **d** Normal phenotype of dividing mitochondrion in cell overexpressing wild-type CmDnm1. **e** Phenotype of single mitochondrion without division in cell overexpressing CmDnm1^S570E^. **f** Phenotype of multiple chloroplasts in CmDnm1^T139A^ overexpression mutant. **g** Frequency of cells with multiple chloroplasts among Dnm1-mutant transfectants. Total countable transfectants were counted. **h** In vitro kinase assay of human Aurora kinases with recombinant GST-human Drp1. Bars: 1 μm.

On the basis of our findings that CmAUR is involved in mitochondrial division and in the phosphorylation of proteins related to mitochondrial division, we hypothesized that CmAUR regulates mitochondrial division via direct phosphorylation of mitochondrial division ring proteins. We predicted that phosphorylation of CmDnm1 by CmAUR would be important for mitochondrial division because CmDnm1, as a component of the mitochondrial division ring, has a crucial role in mitochondrial division in *C. merolae*^4,11^. To further analyze phosphoregulation of CmDnm1 by CmAUR, in vitro phosphorylation sites in CmDnm1 were identified by mass spectrometry (Supplementary Fig. 5). Taking evolutionary conservation among eukaryotes into consideration, nine residues were determined as candidate sites for phosphorylation. Through in vitro kinase assays of point mutants of these nine residues, four phosphorylation sites (T139, S570, S726, S732) were confirmed to be phosphorylated by CmAUR (Fig. 4a). In addition, to confirm each single phosphorylation site, we quantified the western blot signal reduction of phosphorylation site variants. As a result, signal densities of T139A and S726A variants were reproducibly reduced compared with wild-type CmDnm1 signals (Fig. 4b).

To reveal the function of these phosphorylation sites in mitochondrial division, we produced Dnm1 variants in which phosphorylation sites T139, S570, S726, and S732 were substituted to nonphosphorylatable alanine or phosphomimetic glutamic acid and overexpressed them in *C. merolae*. The effects of these variants were quantified by determining the frequency of each mitochondrial division phase in each transformant (Fig. 2d). The population in each mitochondrial division phase was not affected by overexpression of CmDnm1^S570A/E^, CmDnm1^S726A^, or CmDnm1^S732A/E^, but the number of cells in pre-mitochondrial division phase was increased by overexpression of CmDnm1^T139A/E^ and CmDnm1^S726E^ (Fig. 4c; Supplementary Table 4). This result suggests that the expression of CmDnm1^T139A/E^ and CmDnm1^S726E^ causes suppression of mitochondrial division. Previously, it was reported that mitochondrial division is stalled by overexpression of dominant-negative Drp1 mutants (phosphodeficient or mimetic substitution within the GTPase domain)^33^. We observed that overexpression of CmDnm1^T139A/E^ and CmDnm1^S726E^ caused mitochondrial division arrest. These results suggest that T139A/E and S726E function, respectively, in a dominant-negative mode. Interestingly, overexpression of CmDnm1 with T139 variations caused abnormal phenotypes such as mitochondrial distribution on one side of the cell (Fig. 4c–e; Supplementary Table 4) and multiple chloroplasts (Fig. 4f, g; Supplementary Table 5), as described previously^31^. Because aberrant phenotypes were not frequent among the transformants of CmDnm1^S726E^, we predict that phosphoregulation of T139 has a more important role in mitochondrial division than does phosphoregulation of S726.

To investigate whether the phosphorylation of T139 regulates the localization of CmDnm1, we constructed stably transformed lines that expressed superfolder GFP (sfGFP)-CmDnm1, sfGFP-CmDnm1^T139A^, and sfGFP-CmDnm1^T139E^ under the control of a heat-inducible promoter^16^. The heat-inducible expression enables CmDnm1 variant localization to be observed without the effect of a transfection reagent containing polyethylene glycol. After heat induction, we immunostained cells using an Ef-Tu^36^ antibody as a mitochondrial marker and a GFP antibody. We found no differences in the intracellular localization of each CmDnm1 variant (Supplementary Fig. 6). This result indicates that phosphorylation of T139 may regulate CmDnm1 independently from the intracellular localization control.

Finally, we examined if recombinant human Aurora kinase could directly phosphorylate recombinant Drp1, which is a CmDnm1 orthologue found in human. Aurora A and Aurora B could phosphorylate Drp1 in vitro (Fig. 4h). This result suggests that the direct phosphoregulation of CmDnm1 orthologs by Aurora kinase is conserved between *C. merolae* and human.

## Discussion

The results of the present study suggest that CmAUR is involved in mitochondrial division through direct phosphorylation of CmDnm1, the Drp1 orthologue in *C. merolae*. Residues T139 and S726 of CmDnm1 were found to have functional impacts on mitochondrial division in *C. merolae* (Fig. 4c–g).

In this study, CmAUR was found to phosphorylate multiple components of the mitochondrial division machinery in vitro (Fig. 3a–c). These results suggested that CmAUR has multiple roles in regulating mitochondrial division through phosphorylation of multiple targets such as CmDnm1, Mda1, and TOP. In a previous report, overexpression and inhibition of Aurora A resulted in mitochondrial elongation in mammalian cells^37^, suggesting that Aurora kinase has a complex role in controlling mitochondrial morphology. In summary, the complex role of Aurora kinase in mitochondrial division may be conserved in *C. merolae* and mammalian cells.

The nonphosphorylatable mutant CmDnm1^T139A^ and the phosphomimetic mutant CmDnm1^T139E^ exhibited deficient mitochondrial division phenotypes (Fig. 4c–g). This result suggested that residue T139 of CmDnm1 has an important role in mitochondrial division and that strict spatiotemporal control of the switching between phosphorylation and dephosphorylation is necessary for proper mitochondrial division. T139 is located in the GTPase domain (Supplementary Fig. 7). This suggests that T139 phosphorylation may affect the recruitment of GTP to CmDnm1 and GTP hydrolysis.

However, the percentage of the mitochondrial dividing population of CmDnm1^T139A^ and CmDnm1^T139E^-expressing cells in each phase did not seem to be different (Fig. 4c). From this result, we cannot exclude the possibility that the nonphosphorylated threonine residue at position 139 of CmDnm1 is important for the regulation of mitochondrial division and the phenotype on amino-acid substitution to alanine and aspartic acid was an artifact. In order to prove the importance of T139 phosphorylation of CmDnm1 in mitochondrial division, further investigations are needed. In the case of S726 in CmDnm1, a delayed mitochondrial division phenotype occurred only in the phosphomimetic mutant (Fig. 4c). Therefore, we considered that the only function of phosphorylation at S726 is to suppress mitochondrial division. SSDB (Sequence Similarity Data Base) motif research into CmDnm1 suggested that S726 in CmDnm1 is in the GTPase effector domain (GED) (Supplementary Fig. 7). The GED domain in the dynamin family is a component of the stalk which is involved in homodimerization of dynamin^38,39^. Interestingly, in the CmDnm1 orthologs of higher animals and plants, S726 is replaced with acidic amino acids (Supplementary Fig. 8). Therefore, this site is thought to be responsible for a mitochondrial division control mechanism unique to *C. merolae*.

In this study, we discovered that the direct phosphoregulation of CmDnm1 by Aurora kinase is one of the important mechanisms in mitochondrial division in a primitive red alga. The equivalent of T139 in CmDnm1 is well conserved among eukaryotes (Supplementary Fig. 8), and kinase assay using human recombinant Drp1 and human recombinant Aurora kinase indicated the direct phosphorylation of Drp1 by Aurora kinase (Fig. 4h). Moreover, recent study discovered the indirect regulation of mitochondrial division by Aurora kinase^8^ and intramitochondrial localization of Aurora kinase in mammalian cells^37,40^. These facts demonstrate that the relationship between mitochondrial division and Aurora kinase is common to *C. merolae* and other organisms. Therefore, we conclude that the role of Aurora kinase in mitochondrial division is conserved in eukaryotes. These findings suggest that the regulation of mitochondrial division during the cell cycle by a mitotic kinase was acquired in common ancestral cells, and that this function may have contributed to the establishment of endosymbiosis.

## Methods

### Materials

We used *C. merolae* 10D and M4 provided by Professor Tsuneyoshi Kuroiwa.

### Synchronization culture

Synchronized culture was conducted as previously described^41^.

### Immunofluorescence microscopy of *C. merolae*

Immunostaining was performed as previously described^36^. For the primary reaction, CmAUR antibody, phAUR antibody (2914, Cell Signaling Technology, Massachusetts, USA), Ef-Tu antiserum, Mda1 antiserum, α-tubulin antibody (Life Technologies, California, USA), phosphorylated histone H3 Ser10 antibody (Abcam, Cambridge, UK), and GFP antibody (MBL, Aichi, Japan) were used at dilutions of 1:100, 1:20, 1:200, 1:100, 1:600, 1:125, and 1:100, respectively. The secondary antibodies, Alexa 488 and Alexa 546 (Thermo Fisher Scientific, Massachusetts, USA), were used at a dilution of 1:1000. Images were acquired under an upright microscope (BX53, Olympus, Tokyo, Japan) equipped with a DP72 digital camera (Olympus) or a GS3-U3-50S5M-C camera (Point Grey Research, Inc., British Columbia, Canada). The colocalization of the co-stained signals was quantified by EzColocalisation plugin in ImageJ software^46^.

### Immunoblotting

Thiophosphate ester antibody (Abcam), His antibody (MBL) and phospho-histone H3 (Ser10) antibody 05–598 (Merck, Darmstadt, Germany) were used as primary antibodies at dilutions of 1:5000, 1:2000, and 1:7500, respectively. Primary antibodies against CmAUR^24^, phAUR (2914, Cell Signaling Technology), Aurora A (ab13824), and Aurora B (ab45145) (both from Abcam) were used at dilutions of 1:2000. As the secondary antibody, we used anti-rabbit IgG horseradish peroxidase (HRP)-linked species-specific whole antibody (GE Healthcare, Illinois, USA) and anti-mouse IgG (H + L) HRP conjugate (Promega, Wisconsin, USA) at a dilution of 1:10,000. Some membranes were stained with Coomassie Brilliant Blue or colloidal gold^42^ after luminescence reactions to check loading amounts. All original blotting images are shown in Supplementary Fig. 10.

### Sample preparation for western blot of whole cell protein

Synchronized cultured mitotic cells and normally cultured interphase cells were resuspended in lysis buffer (25 mm pH7.5 4-(2-hydroxyethyl)-1-piperazineethanesulfonic acid (HEPES), 150 mm NaCl, 1 mm ethylenediaminetetraacetic acid (EDTA), 1% Triton X-100, 0.1% Na-deoxycholate, 0.1% sodium dodecyl sulfate) supplemented with complete protease inhibitor cocktail (Merck). After incubation for 90 minutes on ice, glass beads were added to the tube, and the tube was shaken using a bead beater (2600 rpm for 1 min). The supernatant of the extracts was used for western blotting.

### Transformation of *C. merolae*

To produce expression vectors for *C. merolae*, the open reading frame (ORF) of CmAUR and the ORF–3′-UTR (140 bp) of Dnm1 were fused with the β-tubulin promoter of *C. merolae* (1955 bp) by polymerase chain reaction using PrimeSTAR HS (TaKaRa, Shiga, Japan). The PCR products were introduced into the multiple cloning sites of pTH2-PL and pMtGFP, respectively. Mutagenesis of CmAUR and Dnm1 was carried out by PCR with KOD plus-Neo polymerase (Toyobo, Osaka, Japan). Primers are shown in Supplementary Table 6. Each plasmid (10 μg) was used to transform *C. merolae* as previously described^15^.

### Production of heat-inducible cell lines and heat induction

To prepare stable *C. merolae* transformants expressing CmDnm1 via a heat shock inducible promoter, linear vector vHS/bt3’ was amplified with primers T139HS(-1)R and T229btUTR(+ 1) (Supplementary Table 7). Template plasmid containing β-tubulin3ʹ-UTR-CmUra-CMD185C-CMD186C-pQE80-CMD184C-heat shock promoter (200 bp upstream of DMJ101C) was kindly provided by Drs. Shin-ya Miyagishima and Takayuki Fujiwara (National Institute of Genetics, Japan).

A sfGFP fragment for expression in *C. merolae*^16^ was amplified using primers HSP15nt_sfGFP_1F and CmDnm1–15nt_sfGFP_714R (Supplementary Table 7), with a plasmid containing *APCC* promoter-*sfGFP-URA*^16^ as the template. A CmDnm1 fragment was amplified using primers sfGFP15nt_CmDnm1_4F and 3UTR15nt_CmDnm1_2307R (Supplementary Table 7), and expression vectors pMtGFP-CmDnm1-WT, pMtGFP-CmDnm1-T139A, and pMtGFP-CmDnm1-T139E as templates. The resultant PCR products were inserted into vHS/bt3’ using the In-Fusion cloning kit (TaKaRa) to produce HSp-sfGFP-CmDnm1(WT, T139A, or T139E) plasmids. Production of stable *C. merolae* cell lines with the heat-inducible gene cassettes was performed as previously reported^16^. Heat induction was performed as described in Supplementary Fig. 9.

### Recombinant protein preparation

Sequences encoding Dnm1, Mda1, and TOP were integrated into the multiple cloning site of pET28-His-TEV. The primers used for cloning are shown in Supplementary Table 6. The plasmids were induced into *Escherichia coli* BL21 and expression of the recombinant proteins was induced by 0.1 mm isopropyl-β-d-thiogalactopyranoside. After cultivation for 6 h at 37 °C, protein-expressing cells were harvested and homogenized by sonication. The insoluble fraction was washed with wash buffer (20 mm Tris-HCl, pH 8.0, 500 mm NaCl, 0.5% Triton X-100) and dissolved in elution buffer (8 m urea, 20 mm Tris-HCl, pH 8.0, 500 mm NaCl). Recombinant GST-CmAUR was prepared with GSTrap HP as previously described^43^. Recombinant human Aurora A (481413, Millipore, Billerica, Massachusetts, USA), Aurora B (A31–10G, SignalChem, Richmond, Canada) and Drp1 (H00010059-P01, Novus, Littleton, Colorado, USA) proteins were purchased.

### In vitro kinase assay

The CmAUR kinase assay was performed as previously described^43^. When immunoblotting was used to detect phosphorylation, ATP was substituted by ATPγS to use the thiophosphate ester for detection. The substrates resolved in elution buffer were used at < 1/60th of the volume of the total reaction solution. The reaction volume was 25 µL. The reaction buffer contained 5 mm HEPES (pH7.5), 50 mm sucrose, 100 mm KCl, 0.1 mm CaCl_2_, 0.4 mm ethylene glycol tetraacetic acid, 1.2 mm ATPγS, and 20 mm MgCl_2_. After the reaction at 30 °C for 30 min, 1.5 μL of 50 mm *p*-Nitrobenzyl mesylate and 2.6 μL of 500 mm EDTA were added to the mixture.

### In vitro CmAUR dephosphorylation assay

Recombinantly expressed CmAUR was incubated with PP1 (P0754, New England Biolabs, Massachusetts, USA). Mammalian phAUR antibody was used to detect time-dependent dephosphorylation of CmAUR. Phosphatase assay was carried out in phosphatase buffer (10 mm Tris-HCl, pH 6.8, 10 mm NaCl, 0.02 mm EDTA, 0.2 mm dithiothreitol (DTT), 0.14 mg/mL bovine serum albumin) at 37 °C for 20 min.

### Mass spectrometry analysis of CmDnm1 phosphorylation

CmDnm1 phosphorylated by CmAUR in vitro as described above was separated on 7.5% TGX gels, and the gels were stained with Bio-Safe Coomassie G-250 Stain. An LTQ-Orbitrap XL (Thermo Fisher Scientific) coupled with an EASY-nLC 1000 (Thermo Fisher Scientific) was used for nano-LC-MS/MS analyses as described previously^44^. The result of this analysis is shown as “Experiment 1” in Supplementary Fig. 5. We also conducted LC-MS/MS analysis with another sample preparation method. The data obtained by the following method is shown in Supplementary Figure 5 as “Experiment 2”. The sample solution from the in vitro kinase assay was dried by evaporation and the residue was dissolved in denaturing solution (8 m urea, 50 mm ammonium bicarbonate, 10 mm DTT). The mixture was incubated at 37 °C for 30 min and then subjected to reductive alkylation treatment with iodoacetamide. Then, 50 mm ammonium bicarbonate was added and the urea concentration in the solution was diluted to 2 m. Finally, trypsin was added, and the mixture was incubated at 37 °C for 16 h. After the reaction, the peptide solution was desalted using a C_18_ Stage Tip. Phosphorylated peptides were fractionated and recovered from the peptide sample, and TiO_2_ resin was used for specific adsorption. The procedure was based on that recommended for the Titansphere Phos-TiO Kit (GL Science, Tokyo, Japan). The eluted sample was dried under reduced pressure, and the residue was dissolved in a mixture of water, acetone, and trifluoroacetic acid (volume ratio 98:2:0.1) and then analyzed by LC-MS/MS. An Ultimate 3000 liquid chromatograph (Nippon Dionex K.K.) and Linear Trap Quadrupole (LTQ) Orbitrap Velos Mass Spectrometer (Thermo Fisher Scientific) were used for LC-MS/MS analysis. Acclaim PreMap100 (Nippon Dionex K.K.) was used as an analytical column. The composition of mobile phase A was water:acetonitrile:formic acid = 98:2:0.1 (volume ratio). The composition of mobile phase B was water:acetonitrile:formic acid = 5:95:0.1 (volume ratio). The gradient of acetonitrile was (minutes, %B, %acetonitrile): (0, 2, 3.86) → (5, 2, 3.86) → (40, 41, 40.13) → (40.01, 95, 90.35) → (45.01, 2, 3.86) → (60, 2, 3.86). The MS scan range was *m/z* 350 to 1,200. The resolution was 30,000. MS/MS data were analyzed with Mascot Daemon ver. 2. 4. 0 (Matrix Science K.K.).

### Statistics and reproducibility

Student’s *t* test and Welch’s *t* test were used for testing the difference in population means. Data sets whose *P* value of *f* test were larger than 0.05 were used Student’s *t* test, and one whose *p* value was < 0.05 were used Welch’s *t* test. For statistical analysis of count data based on mitochondrial morphology, we performed Pearson’s chi-squared test, and the residuals were calculated.

### Reporting summary

Further information on research design is available in the Nature Research Reporting Summary linked to this article.

## Supplementary information

Supplementary Information

Description of Additional Supplementary Files

Supplementary Data 1

Reporting Summary

## Data Availability

The mass spectrometry data were deposited in the jPOST repository^45^ under accession numbers JPST000592 and JPST000543. Full blots are shown in Supplementary Figure 10. Source data are provided in Supplementary Data 1 and all other data are available upon request.
