# Supplementary material for: Cyanidioschyzon merolae aurora kinase phosphorylates evolutionarily conserved sites on its target to regulate mitochondrial division
Source: Commun Biol. 2019 Dec 20;2:477. doi: 10.1038/s42003-019-0714-x (PMC6925296; doi:10.1038/s42003-019-0714-x)
Supplement: Supplementary file 1 — Supplementary Information [file 42003_2019_714_MOESM1_ESM.pdf]

Supplementary Figures

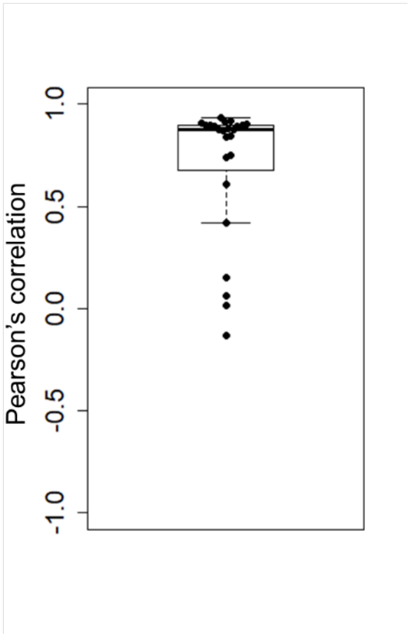

Supplementary Figure 1 Pearson's correlation of CmAUR with mitochondria in *C. merolae*. CmAUR was stained with CmAUR antibody. Mitochondria were stained by Ef-Tu antiserum. Images of immunostained *C. merolae* were analyzed using the EzColocalisation plugin in ImageJ software[46]. For co-localization analysis, plastid areas in each image were eliminated from regions of interest because the autofluorescence of plastids affected our analysis. We used 24 cells for this analysis.

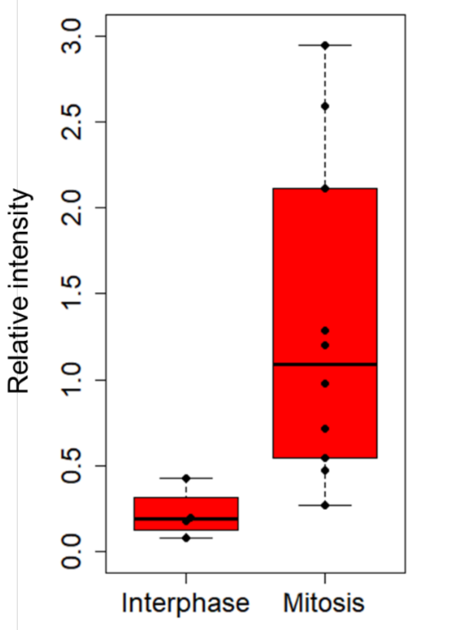

Supplementary Figure 2 Fluorescence intensity of histone H3 Ser10 phosphorylation in *C. merolae*. Fluorescence intensity in *C. merolae* cells, excluding plastid areas, was measured using ImageJ software. Four interphase cells and 10 mitotic cells were analyzed.

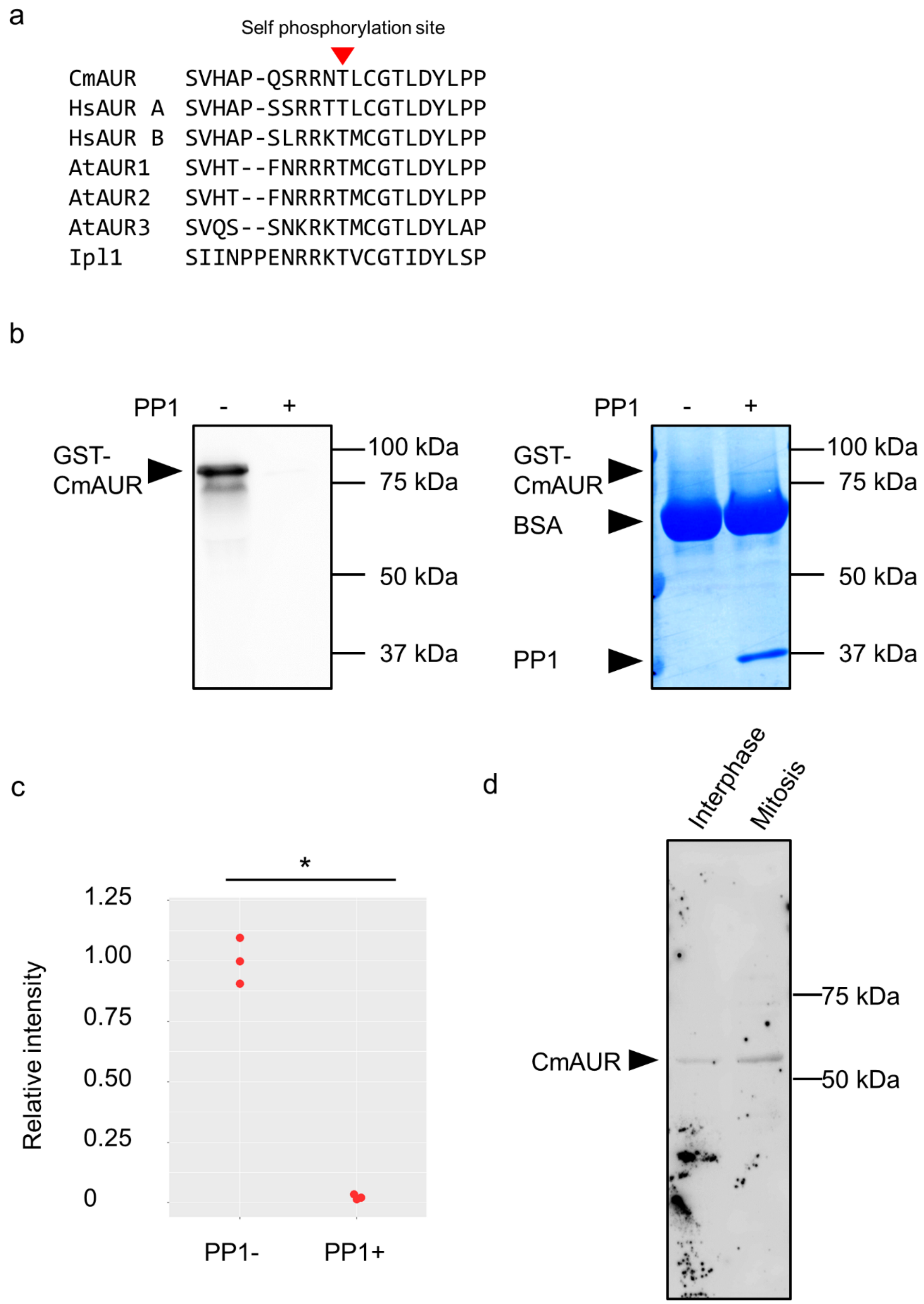

Supplementary Figure 3 Detection of CmAUR self-phosphorylation using mammalian

phosphorylated Aurora kinase antibody.

a, The self-phosphorylation site of Aurora kinase is conserved among eukaryotic organisms. HsAUR, AtAUR, and Ipl1 are Aurora kinases of human, *Arabidopsis thaliana*, and *Saccharomyces cerevisiae*, respectively. **b**, Phosphatase assay of CmAUR protein. Phosphorylated CmAUR was dephosphorylated by incubation with PP1 for 20 min at 37°C *in vitro*. phAUR antibody was used to detect the phosphorylation. **c** Quantification of density in panel **b**. Three independent results were used in this figure. *p = 0.0028 (Welch's t-test). **d** Western blot of whole cell extract of *C. merolae*. Mitotic cells were prepared by light and dark synchronization. Interphase samples were prepared from normal cultured cells. The same number of cells was used for protein extraction, and the same volume of the extracted solutions was loaded onto the gel.

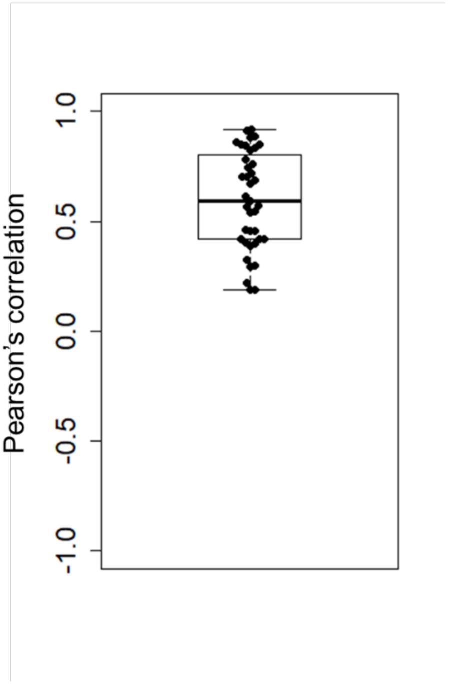

Supplementary Figure 4 Pearson's correlation of phAUR with mitochondria in *C. merolae*. phAUR was stained with phospho-Aurora kinase antibody. Mitochondria were stained with Ef-Tu antiserum. The images of immunostained *C. merolae* were analyzed using the EzColocalisation plugin in ImageJ software[46]. For co-localization analysis, plastid areas in each image were eliminated from regions of interest because the autofluorescence of plastids affected our analysis. We observed 39 cells for this analysis.

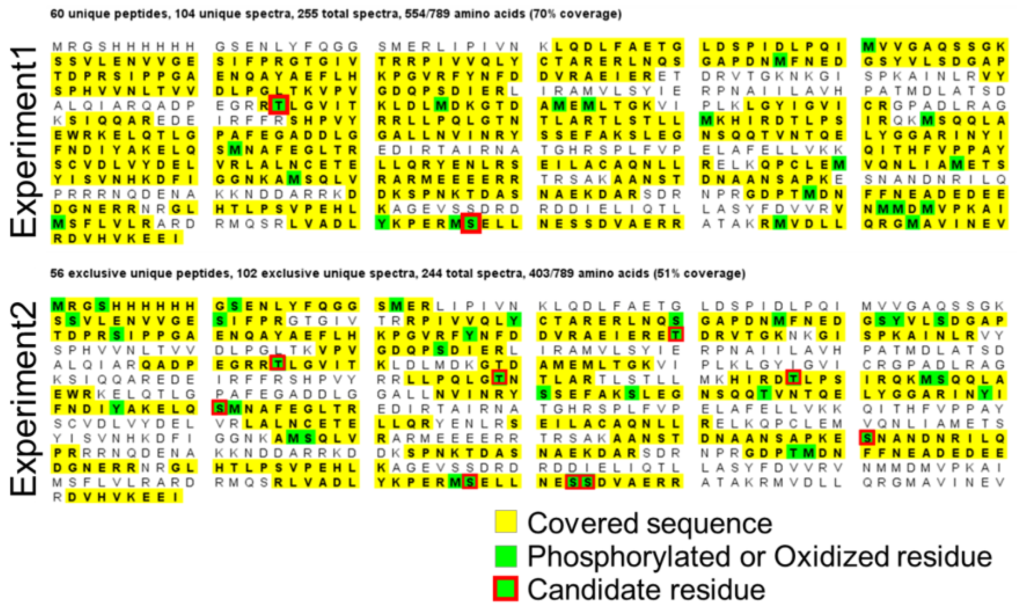

Supplementary Figure 5 Mass spectrometry analyses of CmDnm1 phosphorylated by CmAUR *in vitro*.

A normal peptide accumulation column and a phosphopeptide-specific column were used in Experiment 1 and Experiment 2 respectively. Experiment 1 and Experiment 2 used different approaches, which are explained in Materials and Methods. To briefly explain the difference between the two experiments, in Experiment 1, the phosphorylated CmDnm1 protein was purified by SDS-PAGE before trypsinization. In Experiment 2, the *in vitro* phosphorylation solution was directly trypsinized without electrophoresis. Each of the experiments was performed once.

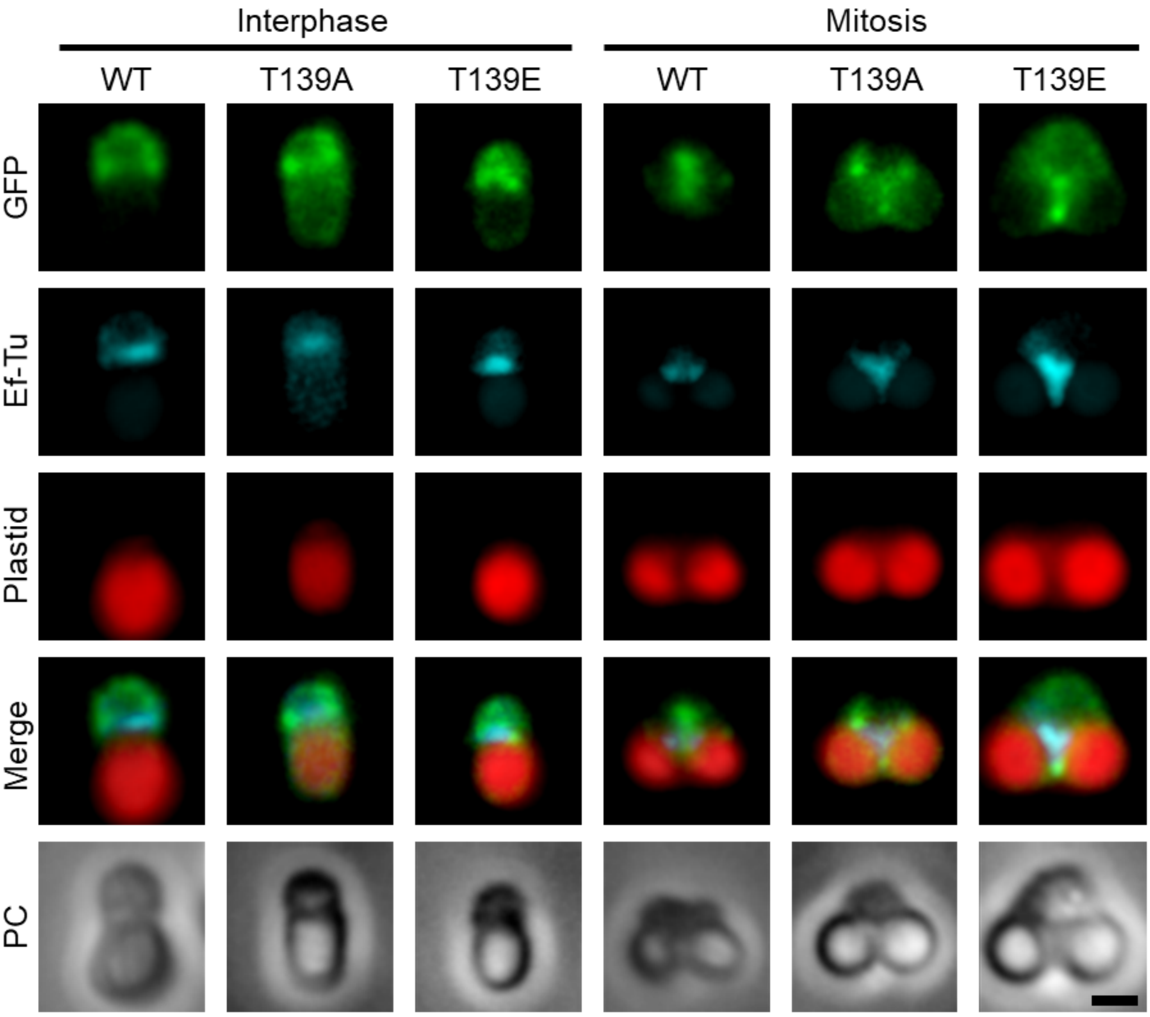

Supplementary Figure 6 Intracellular dynamics of sfGFP-CmDnm1 variants. Representative immunostaining images of CmDnm1-GFP-expressing cells after heat induction. Bar: 1 μm.

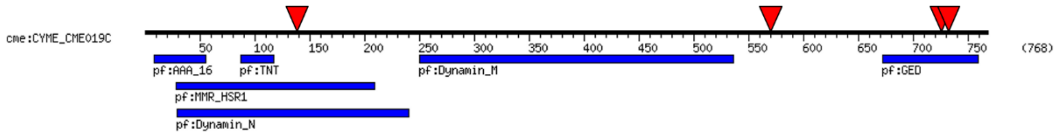

Motif id	From	To	Definition	E value	Score
pf:AAA_16	8	55	AAA ATPase domain	0.2	-
pf:MMR_HSR1	28	209	50S ribosome-binding GTPase	0.0039	-
pf:Dynamin_N	29	240	Dynamin family	4.1e-50	-
pf:TNT	87	117		0.22	-
pf:Dynamin_M	250	536	Dynamin central region	6.5e-95	-
pf:GED	672	759	Dynamin GTPase effector domain	2.1e-23	-

Supplementary Figure 7 Sequence Similarity Data Base motif research result for CmDnm1 (CME019C).

Red arrowheads indicate sites phosphorylated by CmAUR *in vitro*.

Species	Dynamin homologues	Function	Position of N terminus											139											
C. merolae	Dnm1	Mt division	129	D	D	V	R	A	E	I	E	R	E	T	D	R	V	T	G	K	-	N	K	G	I
H. sapiens	DNM1L	Mt division	105	D	E	I	R	Q	E	I	E	N	E	T	E	R	I	S	G	N	-	N	K	G	V
A. thaliana	DRP3A	Mt division	131	S	E	I	R	R	E	I	E	A	E	T	N	R	L	V	G	E	-	N	K	G	V
H. sapiens	Dnm1	Membrane trafficking	95	E	E	V	R	L	E	I	E	A	E	T	D	R	V	T	G	T	-	N	K	G	I
A. thaliana	DL1	Membrane trafficking	101	A	A	V	R	K	E	I	Q	D	E	T	D	R	E	T	G	R	-	S	K	A	I
C. merolae	Dnm2	Pt division	189	A	E	I	Q	A	Y	I	E	A	E	N	L	K	L	E	T	D	P	F	R	S	F
A. thaliana	DRP4A	Pt division	127	E	H	V	A	E	A	I	C	A	A	T	D	V	I	A	G	T	-	G	E	G	V

Species	Dynamin homologues		Position of N terminus											726											
C. merolae	Dnm1	Mt division	718	D	L	Y	K	P	E	R	M	-	-	S	E	L	L	N	E	S	S	D	V	A	E
H. sapiens	DNM1L	Mt division	689	Q	L	Y	K	S	S	L	L	-	-	D	D	L	L	T	E	S	E	D	M	A	Q
A. thaliana	DRP3A	Mt division	715	K	L	Y	R	E	N	L	F	-	-	E	E	M	L	Q	E	P	D	E	I	A	V
H. sapiens	Dnm1	Membrane trafficking	704	N	L	Y	S	C	G	D	Q	-	-	N	T	L	M	E	E	S	A	E	Q	A	Q
A. thaliana	DL1	Membrane trafficking	563	E	L	G	T	M	D	M	K	R	L	S	S	L	L	N	E	D	P	A	I	M	E
C. merolae	Dnm2	Pt division	852	R	L	Y	G	G	D	L	N	D	V	F	N	I	A	E	A	R	A	A	L	E	R
A. thaliana	DRP4A	Pt division		-	-	-	-	-	-	-	-	-	-	-	-	-	-	-	-	-	-	-	-	-	-

Positive Charged
Negative Charged
Polar Uncharged
Special
Hydrophobic

Supplementary Figure 8 Alignment of CmDnm1 variants.

Sequences near sites phosphorylated by CmAUR are shown. Mt and Pt division in the "Function" column represent mitochondrial and plastid division, respectively.

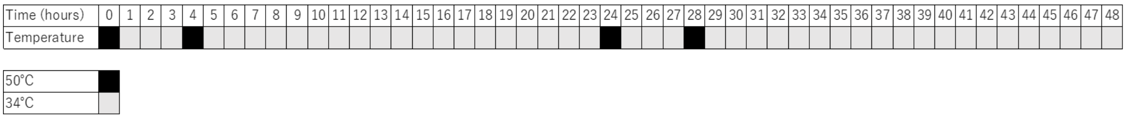

Supplementary Figure 9 Heat induction schedule used in Figure 4i–j. Each heat induction pulse was performed for 1 h. Sampling was conducted 48 h later.

Fig.1

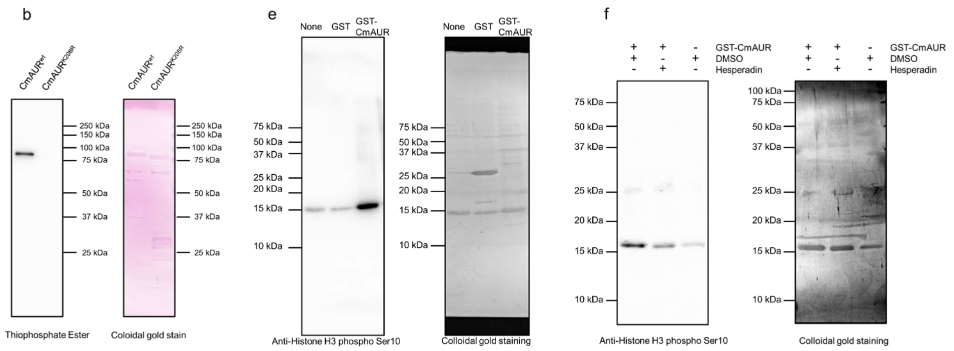

Fig.3

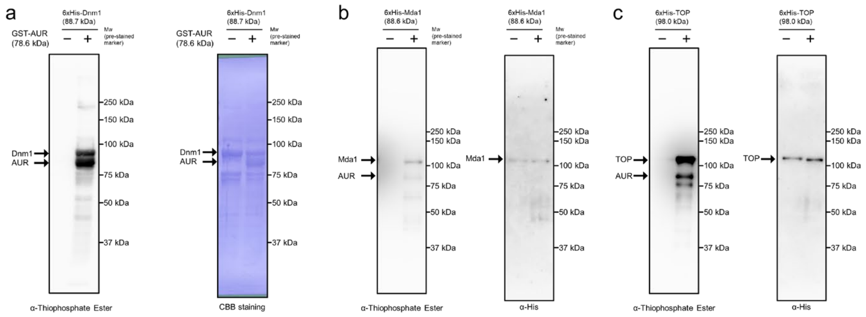

Fig.4

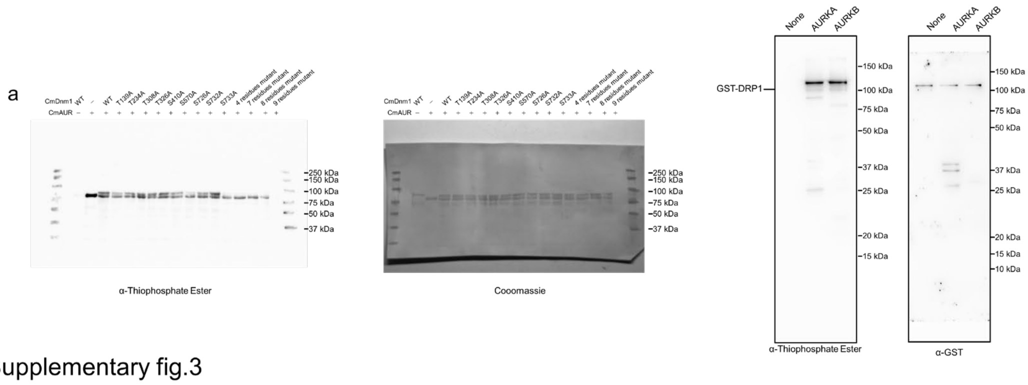

Supplementary fig.3

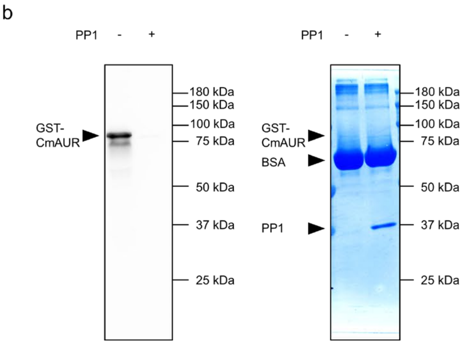

Supplementary Figure 10 All original blotting images from this study.

Supplementary Tables

Supplementary Table 1 Count data for CmAUR^{K208R} transformants.

	Inter	Pre Mt Div.	Post Mt div.
**GFP**	60▲	27▽	19▽
**CmAUR^{208R}-GFP**	13▽	51▲	52▲

$p < 0.01$ (Pearson's chi-squared test). ▲ and ▽ represent a significant increase and decrease in the number of cells, respectively (Pearson's residuals, $p < 0.05$). This experiment was conducted twice.

Supplementary Table 2 Count data for CmAUR-overexpression transformants.

	Inter	Pre Mt Div.	Post Mt div.
**GFP**	100▲	24▽	13▽
**CmAUR-GFP OX**	37▽	54▲	65▲

$p < 0.01$ (Pearson's chi-squared test). ▲ and ▽ represent a significant increase and decrease in the number of cells, respectively (Pearson's residuals, $p < 0.05$). This experiment was conducted twice.

Supplementary Table 3 Mutations in recombinant CmDnm1 (see Figure 4a).

Mutation sites	4 residues mutant	7 residues mutant	8 residues mutant	9 residues mutant
**T139A**	yes	yes	yes	yes
**T234A**		yes	yes	yes
**T308A**		yes	yes	yes
**T326A**				yes
**S410A**			yes	yes
**S570A**	yes	yes	yes	yes
**S726A**	yes	yes	yes	yes
**S732A**	yes	yes	yes	yes
**S733A**		yes	yes	yes

Supplementary Table 4 Count data for CmDnm1 variant transformants.

	Inter	Pre	Post	Failure of Mt div.
**WT**	96 ▲	26 ▽	12	4
**T139A**	27 ▽	30 ▲	5	7
**T139E**	21 ▽	45 ▲	6	12 ▲
**S726A**	92 ▲	33 ▽	12	4
**S726E**	35 ▽	30	17 ▲	0 ▽

$p < 0.01$ (Pearson's chi-squared test). ▲ and ▽ represent a significant increase and decrease in the number of cells, respectively (Pearson's residuals, $p < 0.05$). These data are the sums of two independent experiments.

Supplementary Table 5 Number of wild-type and CmDnm1-variant transformant cells with multiple plastids.

	Normal	Multiple plastids
**WT**	138 ▲	6 ▽
**T139A**	61 ▽	37 ▲
**T139E**	54 ▽	30 ▲
**S726A**	141 ▲	7 ▽
**S726E**	82 ▲	4 ▽

$p < 0.01$; Pearson's chi-squared test. ▲ and ▽ represent a significant increase and decrease in the number of cells, respectively (Pearson's residuals, $p < 0.05$). These data are the sums of two independent experiments.

Supplementary Table 6 Primers used in this study.

Construct	Application of primers	5'→3' Sequence
pCmAUR-GFP	amplification of insert DNA	aggaagcttccttgggatttttctggact
		caagccatggcttgttccgcagcgtgcattc
pProTUB-GFP		gataagcttatccgtactgatgcagacagac
		gtcccatgggacctattctttcttcaccagc
pProTUB-CmAUR-GFP		gataagcttatccgtactgatgcagacagac
		gtcgcctgcatgacctattctttcttcaccagc
		gaataggtcatgcaggcgacaccaggcc
		caagccatggcttgttccgcagcgtgcattc
pET28-TEV-CmDnm1		tatggatccatggagcgcctaatacctatcgtaaac
		ataaagcttctaaatctcctctttgacgtgaacgtc
pET28-TEV-Mda1		tatgaattcatgtttgaggctctttctgggatg
		atagtcgactcactcgggtgtcatgtgcc
pET28-TEV-TOP		tatggatccatgattcgagacagggttccagc
		atagagctcctatggctcctggaaagagactcg
pMtGFP-CmDnm1		gataagcttatccgtactgatgcagacagac
		ggcgctccatgacctattctttcttcaccagcaag
		gaataggtcatggagcgcctaatacctatcgtaaac
		tatggatcccgttcgcagtcgttcgctgc
CmDnm1_T139A	point mutaton induction	aatagaacgagaggcagatcgcgttactggaaaaaacaaggg
		cccttgtttttccagtaacgcgatctgcctctcgttctatt
CmDnm1_T234A		gcgacgcgcgctgggtgtc
		gacacccagcgcgcgtcgc
CmDnm1_T308A		tctgctgccacagctgggagcgaatacgttagcacgaacact
		agtgttcgtgctaacgtattcgctcccagctgtggcagcaga
CmDnm1_T326A		aagcacattcgtgacgcgctgccgagcatacgccag
		ctggcgtatgctcggcagcgcgtcacgaatgtgctt
CmDnm1_S410A		acatttacgcgaaggagctgcaggcgatgaatgcgttcgaa
		ttcgaacgcattcatcgcctgcagctccttcgcgtaaatgt
CmDnm1_S570A		atagtgcaccgaaagaggccaacgcaaacgacaatcg
		cgattgtcgtttgcgttggcctctttcggtgcactat
CmDnm1_S726A		cgaacgtatggcggaactgctcaac
		gttgagcagttccgccatacgttcg
CmDnm1_S732A		tatgagcgaactgctcaacgaggcaagcgacgtagcggagcg
		cgctccgctacgtcgcttgcctcgttgagcagttcgctcata
CmDnm1_S733A		tatgagcgaactgctcaacgagtcagccgacgtagcggagcg
		cgctccgctacgtcggctgactcgttgagcagttcgctcata
CmDnm1_T139E		aatagaacgagaggaagatcgcgttactggaaaaaacaaggg
		cccttgtttttccagtaacgcgatcttcctctcgttctatt
CmDnm1_S570E		atagtgcaccgaaagaggaaaacgcaaacgacaatcg
		cgattgtcgtttgcgttttcctctttcggtgcactat
CmDnm1_S726E		ctacaaacccgaacgtatggaagaactgctcaacgag
		ctcgttgagcagttcttccatacgttcgggtttgtag
CmDnm1_S732E		gaactgctcaacgaggaaagcgacgtagcggagcgac
		gtcgctccgctacgtcgctttcctcgttgagcagttc
CmAUR_K208R		ctacattgtggcactccgtgtcctgttcaaggc
		gccttgaacaggacacggagtgccacaatgtag

Supplementary Table 7 Primers used for the construction of vectors used to produce *C. merolae* lines with heat-inducible CmDnm1.

Primer name	Sequence (5'-3')
T139HS(-1)R	GAATCCCTGGTTCTCTCACAGG
T229btUTR(+1)	TAAACTAGCTATTTATCTGGTACATATCATTCAT
HSP15nt_sfGFP_1F	GAGAACCAGGGATTATGAGCAAGGGCGAGGAGCT
CmDnm1-15nt_sfGFP_714R	AGGTATTAGGCGCTCCTTGTACAGCTCGTCCATGCC
sfGFP15nt_CmDnm1_4F	GACGAGCTGTACAAGGAGCGCCTAATACCTATCG
3UTR15nt_CmDnm1_2307R	TAAATAGCTAGTTTACTAAATCTCCTCTTTGACGTG

Description of additional supplementary items

Supplementary Data1 Source data of this study. The source data underlying plots shown in this paper was provided.
