# Supplementary material for: Cyanidioschyzon merolae aurora kinase phosphorylates evolutionarily conserved sites on its target to regulate mitochondrial division
Source: Commun Biol. 2019 Dec 20;2:477. doi: 10.1038/s42003-019-0714-x (PMC6925296; doi:10.1038/s42003-019-0714-x)
Supplement: Supplementary file 2 — Description of Additional Supplementary Files [file 42003_2019_714_MOESM2_ESM.docx]

**Description of additional supplementary items**

**Supplementary Data1** Source data of this study. The source data underlying plots shown in this paper was provided.
