# Supplementary material for: Cyanidioschyzon merolae aurora kinase phosphorylates evolutionarily conserved sites on its target to regulate mitochondrial division
Source: Commun Biol. 2019 Dec 20;2:477. doi: 10.1038/s42003-019-0714-x (PMC6925296; doi:10.1038/s42003-019-0714-x)
Supplement: Supplementary file 4 — Reporting Summary [file 42003_2019_714_MOESM4_ESM.pdf]

nature research

Corresponding author(s): Sachihiro Matsunaga

Last updated by author(s): Nov 11, 2019

Reporting Summary

Nature Research wishes to improve the reproducibility of the work that we publish. This form provides structure for consistency and transparency in reporting. For further information on Nature Research policies, see Authors & Referees and the Editorial Policy Checklist.

Statistics

For all statistical analyses, confirm that the following items are present in the figure legend, table legend, main text, or Methods section.

n/a	Confirmed	
☐	☒	The exact sample size (*n*) for each experimental group/condition, given as a discrete number and unit of measurement
☐	☒	A statement on whether measurements were taken from distinct samples or whether the same sample was measured repeatedly
☐	☒	The statistical test(s) used AND whether they are one- or two-sided<br>*Only common tests should be described solely by name; describe more complex techniques in the Methods section.*
☒	☐	A description of all covariates tested
☒	☐	A description of any assumptions or corrections, such as tests of normality and adjustment for multiple comparisons
☐	☒	A full description of the statistical parameters including central tendency (e.g. means) or other basic estimates (e.g. regression coefficient) AND variation (e.g. standard deviation) or associated estimates of uncertainty (e.g. confidence intervals)
☒	☐	For null hypothesis testing, the test statistic (e.g. *F*, *t*, *r*) with confidence intervals, effect sizes, degrees of freedom and *P* value noted<br>*Give P values as exact values whenever suitable.*
☒	☐	For Bayesian analysis, information on the choice of priors and Markov chain Monte Carlo settings
☒	☐	For hierarchical and complex designs, identification of the appropriate level for tests and full reporting of outcomes
☒	☐	Estimates of effect sizes (e.g. Cohen's *d*, Pearson's *r*), indicating how they were calculated

Our web collection on statistics for biologists contains articles on many of the points above.

Software and code

Policy information about availability of computer code

Data collection	ImageJ
Data analysis	ImageJ, EzColocalization plugin (ImageJ), R

For manuscripts utilizing custom algorithms or software that are central to the research but not yet described in published literature, software must be made available to editors/reviewers. We strongly encourage code deposition in a community repository (e.g. GitHub). See the Nature Research guidelines for submitting code & software for further information.

Data

Policy information about availability of data

All manuscripts must include a data availability statement. This statement should provide the following information, where applicable:

- Accession codes, unique identifiers, or web links for publicly available datasets
- A list of figures that have associated raw data
- A description of any restrictions on data availability

jPOSR repository (JPST000592 and JPST000543)

Field-specific reporting

Please select the one below that is the best fit for your research. If you are not sure, read the appropriate sections before making your selection.

☒ Life sciences ☐ Behavioural & social sciences ☐ Ecological, evolutionary & environmental sciences

For a reference copy of the document with all sections, see nature.com/documents/nr-reporting-summary-flat.pdf

October 2018

Life sciences study design

All studies must disclose on these points even when the disclosure is negative.

Sample size	We did not use statistical methods to determine sample size. In our study, sample size was determined to be adequate based on the magnitude and consistency of measurable differences between groups.
Data exclusions	No data were excluded without in the case that the control of the experiments did not work.
Replication	Similar results were observed in multiple independent experiments.
Randomization	Materials were randomly picked up for our analyses and data collection.
Blinding	Not applicable.

Reporting for specific materials, systems and methods

We require information from authors about some types of materials, experimental systems and methods used in many studies. Here, indicate whether each material, system or method listed is relevant to your study. If you are not sure if a list item applies to your research, read the appropriate section before selecting a response.

Materials & experimental systems

n/a	Involved in the study
☐	☒ Antibodies
☐	☒ Eukaryotic cell lines
☒	☐ Palaeontology
☒	☐ Animals and other organisms
☒	☐ Human research participants
☒	☐ Clinical data

Methods

n/a	Involved in the study
☒	☐ ChIP-seq
☒	☐ Flow cytometry
☒	☐ MRI-based neuroimaging

Antibodies

Antibodies used	Cyanidioschyzon immunostaining:<br>CmAUR antibody, phAUR antibody (2914, Cell Signaling Technology, Massachusetts, USA), EfTu antiserum, Mda1 antiserum, α-tubulin antibody (Life Technologies, California, USA), phosphorylated histone H3 Ser10 antibody (Abcam, Cambridge, UK) and GFP antibody (MBL, Aichi, Japan)<br>Alexa 488 and Alexa 546 (Thermofisher Scientific, Massachusetts, USA),<br>Western blot:<br>Thiophosphate ester antibody (Abcam, Cambridge, UK) and His antibody (MBL, Aichi, Japan)<br>Aurora A antibody ab13824, Aurora B antibody ab45145 (Abcam, Cambridge, UK), phospho-Histone H3 (Ser10) antibody 05-598 (Merck, Darmstadt, Germany)<br>Horseradish Peroxidase-Linked Species-Specific Whole Antibody (GE Healthcare, Illinois, USA) and Anti-Mouse IgG (H+L), HRP Conjugate (Promega, Wisconsin, USA)
Validation	CmAUR antibody was validated in the study of reference 24. EfTu antiserum was validated in the study of reference 36. Mda1 antiserum was validated in the study of reference 32. The rest of antibodies were purchased from reputable commercial vendors with provided validation information.

Eukaryotic cell lines

Policy information about cell lines

Cell line source(s)	We used C. merolae 10D and M4 provided from Professor Tsuneyoshi Kuroiwa
Authentication	All C. merolae lines were authenticated by the vendor by morphology under the microscope.
Mycoplasma contamination	We have not checked mycoplasma contamination because we are sure that mycoplasma cannot grow in media for C. merolae.
Commonly misidentified lines (See ICLAC register)	None
